# Magnetic Nanoparticles Supporting Bio-responsive *T*_1_/*T*_2_ Magnetic Resonance Imaging

**DOI:** 10.3390/ma12244096

**Published:** 2019-12-07

**Authors:** Connor M. Ellis, Juan Pellico, Jason J. Davis

**Affiliations:** Department of Chemistry, University of Oxford, South Parks Road, Oxford OX1 3QZ, UK; connor.ellis@chem.ox.ac.uk (C.M.E.); juan.pellico@kcl.ac.uk (J.P.)

**Keywords:** Bio-responsive, Nanoparticle, pH-responsive, Enzyme-responsive, Biomolecule-responsive, Magnetic Resonance Imaging, Diagnosis, Therapy, Mesoporous Silica Nanoparticles, Iron Oxide Nanoparticles

## Abstract

The use of nanoparticulate systems as contrast agents for magnetic resonance imaging (MRI) is well-established and known to facilitate an enhanced image sensitivity within scans of a particular pathological region of interest. Such a capability can enable both a non-invasive diagnosis and the monitoring of disease progression/response to treatment. In this review, magnetic nanoparticles that exhibit a bio-responsive MR relaxivity are discussed, with pH-, enzyme-, biomolecular-, and protein-responsive systems considered. The ability of a contrast agent to respond to a biological stimulus provides not only enriched diagnostic capabilities over corresponding non-responsive analogues, but also an improved longitudinal monitoring of specific physiological conditions.

## 1. Introduction

**Figure 1:**
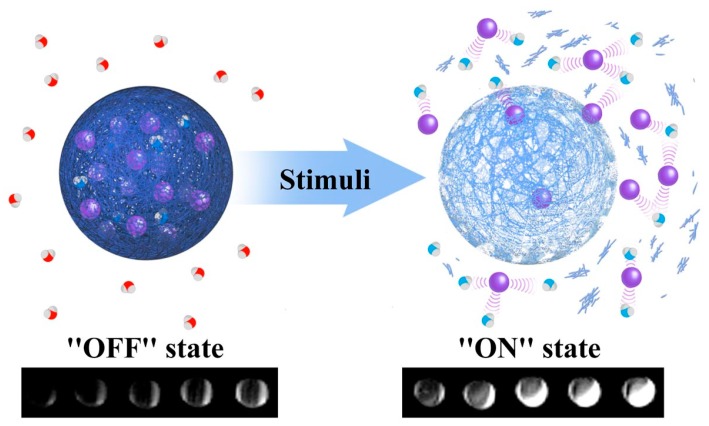
A schematic representing the impact of a generic stimulus on nanoparticle generated magnetic resonance imaging (MRI) contrast. The stimulus may be pH, enzyme activity, or temperature in nature, or may reflect the prescence of specific proteins/enzymes. In this example *T*_1_ contrast capabilities are switched “on”/“off” with a particular biological stimulus. The MR active moieties (purple spheres) are encapsulated within a responsive matrix and released into solution in the prescence of this particular stimulus (enhancing MR contrast by interacting with local water proton; red = non-enhanced relaxation, blue = enhanced relaxation). Adapted with permission from the authors of [13]. Copyright (2013) American Chemical Society.

Medical imaging is one of the most important tools available to a physician seeking to both diagnose and crystallise a prognosis. The modalities available for this are broad, primarily including magnetic resonance imaging (MRI), positron emission tomography (PET), computed tomography (SPECT), X-ray, and ultrasound (US). Of these, MRI constitutes the most convenient way of generating pathological detail at a deep tissue depth. Though now routine, MRI is inherently insensitive in generating contrast at specific incident imaging fields [1]. The use of a contrast agent during an MRI scan dramatically improves the sensitivity by interacting with local water protons and shortening their relaxation times [2,3,4]. The high surface area to volume ratio and chemical tunability of nanoparticles lead to high levels of potential functionalisation, with, for example, an improved biocompatibility upon tethering with polymers such as poly(ethylene glycol) (PEG) [2]. The sizes of particles can be modified to influence their route of clearance from the body, with small particles (<7 nm) rapidly cleared from the body via the kidney glomerulus. Nanoparticles that are larger in size are cleared by the reticuloendothelial system (RES), with the surface chemistry of the nanoparticles easily tuned to increase blood circulation times on chemical tethering. This provides a general marked improvement in relaxometric properties in comparison with their molecular analogues [5]. Biocompatible polymers, such as PEG, are used for this process, promoting a greater acquisition time and, hence, image quality. The ability of a contrast agent to respond to a specific biological stimulus is an invaluable diagnostic asset, with many diseases generating localised conditions that deviate from the standard background [6]. This can be accomplished by manipulating the sizes of particles to switch between the mode of contrast with the formation of nanoaggregates, as exemplified by pH-sensitive iron oxide systems. In addition, the choice of ligand can affect the responsive behaviour, where diffusive water access to the paramagnetic site can be manipulated by, for example, stimuli-responsive polymers such as poly(acrylic acid), poly(L-histidine), or chitosan [7,8,9,10]. Alternatvely, molecular binding proteins can be incorporated onto the nanoparticle scaffold, promoting changes in contrast capabilities on recognition of a specific target, such as dopamine [11]. There has accordingly been much effort invested in the generation of contrast agents responsive to deviations in pH, biomolecular distribution, and enzyme activity (Table 1) [12,13]. These efforts will, therefore, provide the focus of this brief review. Physiologically-responsive MRI contrast agents are designed to switch “on”/“off” in response to a specific stimulus, as seen in Figure 1. In this example, there is a clear brightening of the phantom from the “off” state to the “on” state, thus providing an example of a *T*_1_ responsive system. Alternatively, the system may be *T*_2_ responsive, with an associated darkening in MRI contrast on stimulation or the agent may indeed be dual *T*_1_/*T*_2_ responsive, where the dominant mode switches depending on the local environment.

## 2. MRI Theory

Water molecules are abundant within tissues, differing in concentration depending on the particular environment in which they reside. MRI operates by mapping the spatial distribution of the water protons to generate a 3D anatomical image of the pathological region of interest. In order to understand how effective a particular contrast agent is, one quantifies its concentration-dependent ability to shorten the longitudinal/transversal relaxation rates of water protons. If an external magnetic field is applied, the individual water proton spins align either parallel or antiparallel with the magnetic field, generating a net magnetisation along the principal, or longitudinal, axis. If a radiofrequency pulse is applied, this net magnetisation rotates away from the longitudinal axis into the perpendicular plane (inducing a net magnetisation). On removal of this pulse, the net magnetisation is recovered to the equilibrium state by two distinct processes, termed *T*_1_ and *T*_2_ relaxation. *T*_1_, or longitudinal relaxation, characterises the recovery of the net magnetisation along the longitudinal axis, while *T*_2_, or transverse relaxation, is the recovery within the transverse plane. The relaxation process that a particular contrast agent promotes has implications on the type of contrast generated, with *T*_1_ contrast agents brightening the physiological region of interest and *T*_2_ darkening this region. The relaxation rate (*R_i_*) can be calculated as follows:

(1) $$R_{i}=\frac{1}{T_{i}}\;\;\;\;\;\;\;\;\;i=1,2,$$

where *R_i_* corresponds to the relaxation rate, with the relaxation rate in the longitudinal axis denoted *R*_1_ and that in the transverse plane denoted as *R*_2_. This can then be used to calculate the relaxivity (*r_i_*) if the concentration of the contrast agent is known:

(2) $$r_{i}=\frac{\Delta R_{i}}{c_{\mathrm{CA}}}\;\;\;\;\;\;\;\;\;\;\;\;\Delta R_{i}=\left[\frac{1}{T_{i,\;\mathrm{obs}}}-\frac{1}{T_{i,\;\mathrm{water}}}\right]\;\;\;\;\;\;\;i=1,2,$$

where *r_i_* is the relaxivity, Δ*R_i_* is the change in the relaxation rate between the observed and native water relaxation rates, and *c*_CA_ is the concentration of contrast agent. When *i* = 1, the longitudinal relaxivity is being considered, and the transverse relaxivity when *i* = 2. It is often useful to determine the relaxometric ratio (*r*_2_/*r*_1_) for a given contrast agent, because a larger relaxometric ratio corresponds to a greater *r*_2_ contribution, and hence a more effective *T*_2_ agent, with the reverse true for a *T*_1_ agent.

Production of an effective stimuli-responsive contrast agent needs careful consideration of the parameters that govern relaxivity. The most significant factors affecting any switch in relaxivity are the hydration number (*q*), the rotational correlation time (*τ*_R_), and the water residency time (*τ*_M_) [14]. In general, the relaxation rate can be split into both inner sphere and outer sphere contributions (to the relaxivity), with the former directly proportional to *q* and given by the following equation:

(3) $$R_{1}^{IS}=\frac{\left[CA\right]q}{55.6}\left(\frac{1}{T_{1m}+\tau_{M}}\right),$$

where $R_{1}^{\mathrm{IS}}$ is the inner sphere contribution to the relaxation rate, [CA] is the concentration of contrast agent, and *T*_1m_ is the longitudinal relaxation of the bound water to the contrast agent, with all other quantities previously defined [15]. As can be seen from Equation (3), increasing *q* will give a greater relaxation rate, and thus relaxivity. Commonly metal chelates are utilised as MRI probes as encorporation of the metal ion eliminates the associated toxicity of the free ion. By lowering the denticity of the metal associated chelate, *q* values can be increased, but this should be weighed against the loss in thermodynamic stability [14]. Ideally, the water residency time, *τ*_M_, should be much shorter than the longitudinal relaxation of bound water for efficient relaxation (*τ*_M_ < *T*_1m_), but not so short that the water molecules do not undergo full relaxation on interacting with the paramagnetic chelate (otherwise exchange will take place before relaxation). Optimal vales are usually found in the order of 10 ns [16,17]. In addition to inner-sphere contributions, second-sphere and outer-sphere contributions are also important, with the distance of closest approach of outer-sphere water molecules (*a*) and the diffusional correlation time of outer-sphere water molecules (*τ*_D_) influencing relaxivity [16]. A key contribution to relaxivity is the water access to the MR active site. This can be integrated into a designed responsive agent where, for example, stimuli-responsive polymers are incorporated into a nanoparticulate scaffold, with associated conformational changes in the polymer directly impacting water access to the paramagnetic centre (decreasing direct water access to the metal-chelate lowering the relaxivity). Increases in *τ*_R_ (slowing rotational motion) can be influenced by a change in the contrast agent aggregation state [18]. This is exemplified in work with iron oxide nanoparticles (IONPs), where irreversible changes in aggregation state cause a switch in contrast capabilities (between *T*_2_ and *T*_1_), as discussed in the next section of this review.

## 3. Bio-Responsive Magnetic Nanoparticles

### 3.1. pH-Responsive

The most common class of bio-responsive magnetic nanoparticles are those that respond to a change in local pH. Deviations in pH are prevalent in a wide variety of diseases, for example, cancer, inflammation, and Alzheimer’s are all associated with decreased pH levels in local tissues [19,20]. pH-responsivity is achieved by several adopted approaches based largely on modulating water access to magnetic centres through, for example, the use of acid labile bonds (e.g., hydrazone linkages), pH-sensitive polymers (e.g., poly(acrylic acid) (PAA)), or structural decomposition by a disassembly process or degradation [21,22,23,24].

Iron oxide nanoparticles (IONPs) have been heavily employed in this context, with water access and aggregation switching being the primary responsive feature [25,26]. One such example involves the use of commercially available IONPs, where the carboxymethyl dextran coating has been loaded with doxorubicin (DOX) via electrostatic interactions [26]. Upon protonation of the dextran polymer in an acidic environment, the attractive electrostatic interactions between the positively charged DOX and the polymer are turned off, resulting in DOX release. The DOX presence was proposed to hinder the diffusion of water molecules within the coating of the particle, such that its release was then associated with greatly enhanced *T*_1_ and *T*_2_ relaxivities. As DOX is a chemotherapeutic agent, configurations such as this enable a dual-modal diagnosis and treatment.

**Figure 2:**
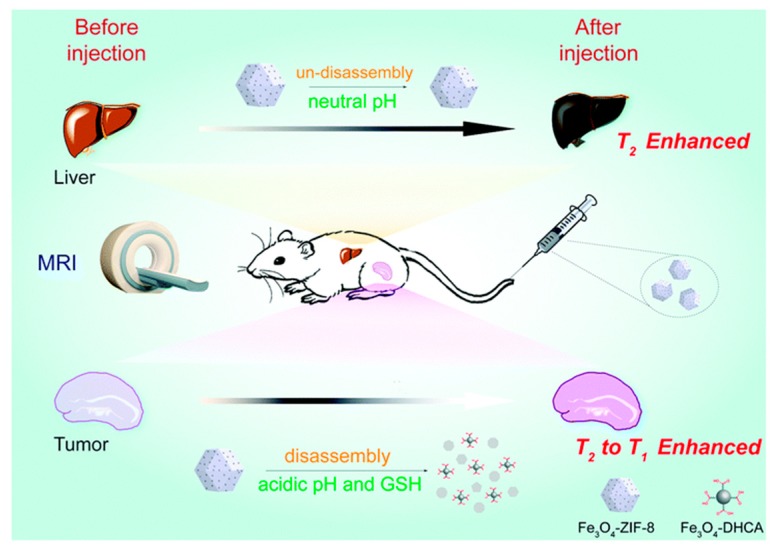
A schematic showing how the incorporation of iron oxide nanoparticles (IONPs) into a pH-responsive zeolitic imidazole framework (ZIF-8 moiety) can engender a responsive contrast capability. At neutral pH, the nanocomposite remains intact, with *T*_2_ contrast exhibited. On decreasing pH, the ZIF-8 structure disassembles switching from *T*_2_ to *T*_1_ contrast capabilities. This framework was shown to be similarly responsive to glutathione (GSH). Reproduced from the work of [30] with permission from The Royal Society of Chemistry.

A manipulation of IONP aggregation state can, as noted, also be exploited. Ordinarily, IONPs are *T*_2_ active, however, the synthesis of small IONPs (< 20 nm) reduces this *T*_2_ activity (and their saturation magnetisation) because of small magnetic domains within the particle [27,28,29]. Consequently, they function as *T*_1_ contrast agents. The formation of aggregates within solution, however, results in a nanocomposite with a high saturation magnetization, and thus *T*_2_ activity. One such example of such individual particle-nanocomposite switching has been through the incorporation of IONPs into a zeolitic imidazole framework (ZIF) to produce an Fe_3_O_4_-ZIF-8 assembly [30]. These disassemble under an acidic environment (pH < 6.2, Figure 2), releasing the individual IONPs, which function as a *T*_1_ agent, whilst the initial Fe_3_O_4_-ZIF-8 assembly is *T*_2_ active. Consequently, disassembly of the framework results in the irreversible switch from negative to positive contrast. The relaxometric ratio (*r*_2_/*r*_1_) for this system was specifically observed to decrease from *r*_2_/*r*_1_ = 24.6 at pH 7.4 to *r*_2_/*r*_1_ = 5.7 at pH 5.0 (indicating a strong progression towards *T*_1_ performance). The same assemblies were also shown to disassemble in the presence of glutathione (overexpressed within the tumour site), indicating additional redox-responsiveness. Another method of incorporating IONPs into a nanocluster assembly relies on an i-motif linker [31]. I-motif DNAs undergo a structural change with pH, causing dissociation of the nanocluster assembly under conditions of low pH. Again, a change in contrast capabilities from *T*_2_ to *T*_1_ is observed as individual IONPs are released, with the relaxometric ratio decreasing significantly from *r*_2_/*r*_1_ = 63.3 at physiological pH to *r*_2_/*r*_1_ = 7.1 at pH 5.5. The self-assembly of IONPs containing pH-responsive ligands has similarly been reported to produce pH-sensitive magnetic ‘nanogrenades’ (PMNs) [32]. These contain imidazole functional groups providing pH-sensitivity (imidazole p*K*_a_ ~ 6.8), catechol groups to facilitate the self-assembly (catechol having a high affinity for the IONP surface), and phenyl entities to control the hydrophobicity of the particles. Within a tumour microenvironment (~pH 6.8), the imidazole groups protonate and electrostatically repel, the hydrophobic interactions weaken, and complete dissociation into individual IONPs occurs. This process is accompanied by a markedly more efficient *T*_1_ relaxation. A similar approach has also been applied using hydrazine cross-linked IONP nanocluster assemblies [25]. As pH decreases, the hydrazone linkages hydrolyse (pH 5.5), releasing individual IONPs, and greatly amplifying the *T*_1_ contrast. This is reported in work by Li et al., where *r*_2_/*r*_1_ = 33.8 at physiological pH and 4.2 at pH 5.5 [25].

Mesoporous silica nanoparticles (MSNs) were first reported in 1992, building on the original synthesis of silica nanoparticles by Stöber et al. in 1968 [33,34]. The mesoporous nature, achieved by the use of a surfactant template, paved the way for their use in a vast range of applications, including contrast agents for drug delivery, multi-modal imaging, and MRI [35,36,37]. The latter has been achieved by doping the MSNs with paramagnetic ions, often by functionalisation with Gd-containing macrocycles [38].The porous nature is attractive, as functionalisation of Gd-chelates in the outer pore channel produces nanoparticles with greater relaxivities than corresponding non-porous analogues. This has been ascribed to the confinement effects around the Gd-chelate that favourably reduce water exchange/tumbling rates, while retaining facile access to the bulk water. By further expanding this platform, bio-responsivity can be accomplished, with one of the first reported examples of this using MSNs in combination with external surface protein recognition to reversibly gate *T*_1_ contrast capabilities [39].

**Figure 3:**
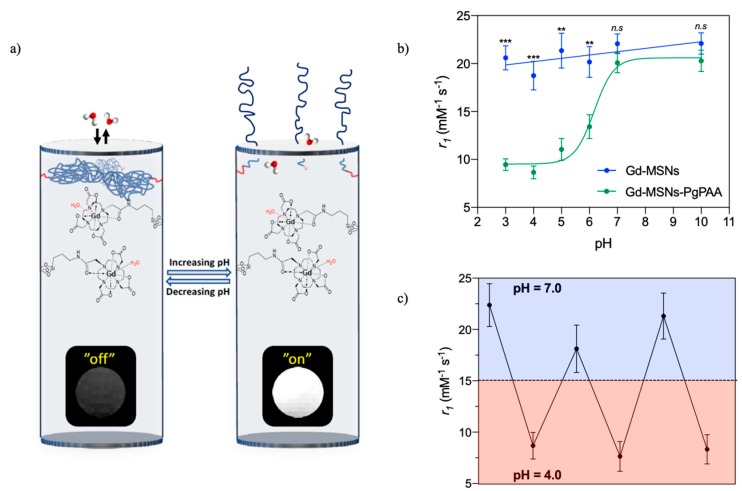
(**a**) Schematic representing a pH-responsive, reversible capping of the pore channels of a mesoporous silica nanoparticle (MSN) with poly(acrylic acid) (PAA) (blue polymer chains). The associated change in MR contrast is also shown. (**b**) A graph detailing the change in relaxivity with pH. (**c**) The reversible relaxivity switching with pH. Adapted from the work of [40] with permission from The Royal Society of Chemistry.

The functionalisation of MSNs with polymers containing ionisable groups introduces a generic pH-responsivity that has been utilised in gatekeeper switching based drug release. Polymers such as poly(vinyl pyridine) (PVP), chitosan, poly(acrylic acid) (PAA), and poly(L-histidine) (PLH) have all been applied on MSNs for this purpose [7,8,9,10]. Recently, Gd-chelates have been incorporated into MSNs capped with the pH-responsive polymer PAA to produce a pH-responsive nanoparticulate *T*_1_ contrast agent (Figure 3) [40]. The pH-responsivity here arises from the protonation state of the monomer unit and associated polymer conformational change from a hydrophobic, collapsed conformation to a hydrophilic, extended conformation as it charges. With the polymer in its extended state, the improved water access through the pore channels leads to a reversible 130% increase in *r*_1_ values (Figure 3c). This work constitutes the first reported example of a fully reversible pH-responsive nanoparticulate *T*_1_ contrast agent.

The integration of manganese oxide into MSNs can also be used to produce *T*_1_ active particles. If embedded in hollow MSNs, the manganese oxide can be reduced in an acidic environment releasing paramagnetic, *T*_1_ active Mn^2+^ ions [41]. These particles have been shown to exhibit an 11-fold increase in relaxivity as solution pH decreases from pH 7.4 to pH 5.0. In addition, the hollow MSN scaffold provides a multi-modal imaging ability, as the hollow interior is effective at amplifying sound, and thus returning an ultrasound signal. Manganese has also been combined with the IONP scaffold to produce pH-sensitive contrast agents. This is exemplified in work by Duan et al., where MnO_2_ nanosheets were imbedded into carbon coated IONPs to produce dual-modal *T*_1_/*T*_2_ magnetic nanoparticles [42]. IONPs within the core support *T*_2_ contrast acquisition, whilst the degradation of the MnO_2_ nanosheets in an acidic microenvironment (releasing Mn^2+^) promote a switch to a dominant *T*_1_ response.

### 3.2. Biomolecule-Responsive

**Figure 4:**
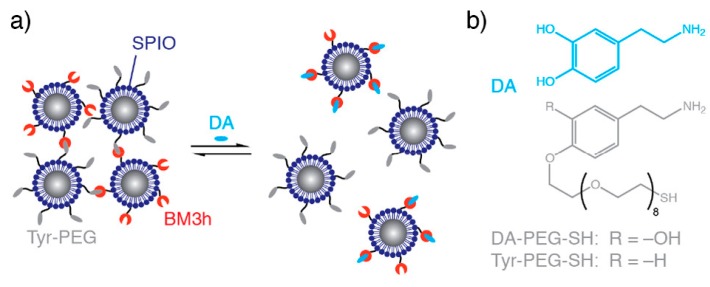
Dopamine sensitive IONPs. (**a**) The effect of dopamine on particle aggregation. In the absence of dopamine, nanocluster assemblies form owing to binding between protein (BM3h) and a dopamine analogue (Tyr-PEG). On addition of dopamine (DA), competitive binding inhibits self-assembly. (**b**) The structures of dopamine and tethered dopamine analogue. Reprinted (adapted) with permission from the authors of [11]. Copyright (2019) American Chemical Society.

An image contrast responsive to the presence of specific biomolecules or ions can be a potent means of imaging localised biological activity. For example, Ca^2+^ is important in cell signalling and Zn^2+^ is vital in cellular physiology [43,44]. Neurotransmitters, such as serotonin, dopamine, oxytocin, and acetylcholine, are vitally important in regulating chemical communication within the brain and have also been of focus in responsive imaging [11,45]. The development of IONPs for which the magnitude of the transversal relaxivity responds to dopamine and serotonin has, for example, been reported by Hsieh et al. (Figure 4) [11]. In this work, two types of IONPs were synthesised, one functionalised with a dopamine binding protein (BM3h), and the other an analogue with the ability to compete with dopamine for protein binding (Tyr-PEG). In the absence of dopamine, these complementary IONPs bind together, forming clusters of particles with a high *r*_2_ value. However, in the presence of dopamine, a potent competitor, inter-particle binding is impaired, resulting in a disassembly of the composite and an associated decrease in *r*_2_. A similar architecture, modified for a serotonin response, was additionally developed. The same group also reported Ca^2+^ sensitive IONPs that undergo a similar, but reversible aggregation mediated by an IONP surface coating with synaptotagmin. The synaptotagmin monomer has two binding sites for Ca^2+^, with IONP aggregates forming in the prescence of Ca^2+^ promoting improved *T*_2_ contrast abilities over the individual IONPs. These particles were proposed as a candidate for mapping brain activity, because changes in Ca^2+^ concentration are indicative of synaptic activity [46]. 

### 3.3. Protein-Responsive

A subset of biomolecule-responsive contrast agents are those that have the ability to respond to a specific protein, a potent potential asset in, for example, cancer diagnostics, where tumour growth is often associated with an overexpression of potential marker proteins [47,48]. In proof of principal work, protein recognition has been achieved with biotin surface modified, Gd-doped MSNs, reversibly switching *T*_1_ contrast through a reversible steric capping of the pore channels with streptavidin [39]. On binding streptavidin, successful capping of the pore channel was achieved, inhibiting diffusive water access to the internal paramagnetic chelates and rendering low relaxivity. In the prescence of excess biotin in solution, streptavidin was observed to be competed off the surface of the particle, restoring water access and high relaxivity.

**Figure 5:**
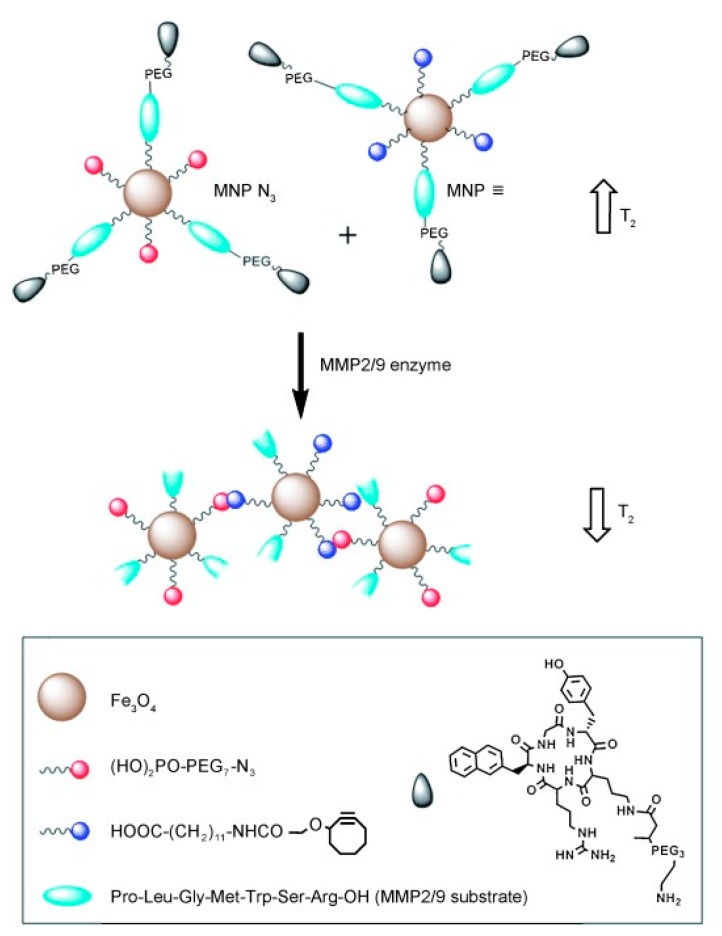
A schematic representing the click coupling of IONPs functionalised with azide (red) groups and those functionalised with alkyne groups (blue). These particles expose azide/alkyne groups in the prescence of matrix metalloproteinase (MMP) enzymes, facilitating a click reaction mediated particle coupling. The effect on the *T*_2_ relaxation can be seen alongside both the individual IONPs (top) and the nanocluster (bottom). The particles also contain tumour specific peptide ligands and are functionalised with PEG to improve biocompatibility *in vivo*. Reprinted with permission from the authors of [52]. Copyright (2014), with permission from John Wiley and Sons.

The role of enzymes in biological and metabolic conditions is also well established, where the presence of specific forms can be associated with a range of pathological conditions, for example, cancer, inflammation, or neurodegeneration [49,50,51]. Often, enzyme-responsive contrast systems contain cleavable linkages that alter the relaxometric properties of the nanoparticulate platform. One such example is the sensing of matrix metalloproteinase (MMP) enzymes by IONPs [52]. In this work, two different particles were produced, one functionalised with azide groups, and the other with alkyne groups, with both containing peptide sequences that have the ability to target the specific tumour site. In the presence of MMP enzymes, the peptide sequence is cleaved, exposing the alkyne/azide groups on the particles, facilitating a “click” reaction between the two, forming nanocluster assemblies. These display a greater saturation magnetisation than the individual IONPs, accompanied with a 160% increase in *T*_2_ activity (Figure 5). Alternatively, nanoparticles with the ability to mimic enzymatic activity, in particular MnO nanoparticles that are able to imitate superoxide dismutase (SOD) enzymes, have recently been reported [53]. Tumour tissues often show elevated concentrations of superoxide radicals, as the enzyme responsible for regulating their level, SOD, is present in lower quantities within the cells. The MnO particles were observed to be reduced by the superoxide radicals, inducing a decrease in the electronic spin of the metal ion, and thus a longer relaxation time.

## 4. Conclusions and Future Outlook

Nanoparticulate MR contrast agents offer improved blood circulation times and markedly greater relaxivities compared with molecular analogues, and can be readily designed to integrate both additional imaging modes and a responsive drug delivery capability. By further introducing bio-responsivity, these become more potent still and one can foresee their use becoming ever more prevalent in early identification of disease. This bio-responsivity arises from the chemists’ ability to understand and tune characteristics that govern relaxivity; for example, the manipulation of diffusive water access to magnetic centres or of rotational motion by controlling aggregation. Within this short review, nanoparticles with pH-, enzyme-, molecular-, ion-, and protein-responsive properties have all been discussed and exemplified with recent works. Despite much progress during the past decade, there remains an enormous scope to advance this field, with a plethora of physiological conditions that could be investigated. One potentially fruitful organic–inorganic marriage is, for example, the integration of thermo-responsive polymers with paramagnetic/superparamagnetic metal hosting particle architectures. These polymers can mediate a significant change in hydration and solubility with changes in temperature [54,55,56], and have been applied to thermo-responsive drug release [57,58,59,60]. The integration of an associated high resolution imaging mode, such as MR, would also facilitate a direct imaging of treatment efficacy by magnetic hyperthermia. In addition, the versatility of nanoparticulate platforms provides scope to introduce additional imaging modalities, such as the incorporation of radiotracers to produce dual-modal PET/MR-responsive agents. MSNs represent one such promising platform, as amino anchor groups can be introduced with relative ease, facilitating a broad range of subsequent functionalities to be tethered. Furthermore, therapeutic agents can be loaded within porous or hollow particles such as these and thus produce a potent multimodal, bio-responsive theranostic agent. The application of engineerable platforms such as this could revolutionalise the way disease states are diagnosed, monitored, and treated.

## Figures and Tables

**Table 1:** A summary of the bio-responsive magnetic nanoparticulate systems encompassed in this review. The sizes, changes in relaxivity, stimulus, as well as the advantages of each sytem are included. MMP, matrix metalloproteinase; PAA, poly(acrylic acid); MSN, mesoporous silica nanoparticle; MRI, magnetic resonance imaging; GSH, glutathione.

Type	Size	Relaxivities	Stimulus	Advantages	Reference
Hydrazine cross linked iron oxide nanocluster assemblies	Iron Oxide Nanoparticles = 9 nm, Nanocluster = 60 nm	*r*_2_/*r*_1_ = 4.2 at pH 5.5, *r*_2_/*r*_1_ = 33.8 at pH 7.4	pH	*T*_1_/*T*_2_ switch in contrast capabilities	[25]
Fe_3_O_4_-ZIF-8 assemblies	Iron Oxide Nanoparticles = 15 nm, Fe_3_O_4_-ZIF-8 = 120 nm	*r*_2_/*r*_1_ = 5.7 at pH 5.0, *r*_2_/*r*_1_ = 24.6 at pH 7.4	pH, GSH (redox)	*T*_1_/*T*_2_ switch in contrast capabilities	[30]
i-motif DNA-assisted iron oxide nanocluster assemblies (RIAs)	Iron Oxide Nanoparticles ~3 nm, RIAs = 120 nm	*r*_2_/*r*_1_ = 7.1 at pH 5.5, *r*_2_/*r*_1_ = 63.3 at pH 7.4	pH	*T*_1_/*T*_2_ switch in contrast capabilities	[31]
pH sensitive ‘magnetic nanogrenades’ (PMNs)	Iron Oxide Nanoparticles ~3 nm, PMNs ~60 nm	*r*_2_/*r*_1_ = 5.8 at pH 5.5, *r*_2_/*r*_1_ = 13.3 at pH 7.4	pH	*T*_1_/*T*_2_ switch in contrast capabilities	[32]
Biotin surface modified Gd-doped MSNs	75 ± 6 nm	Native MSNs *r*_1_ = 15.1 mM^−1^ s^−1^, Biocapped MSNs *r*_1_ = 5.8 mM^−1^ s^−1^	Biotin	Reversible protein recognition	[39]
Gd-MSNs-PgPAA	61 ± 8 nm	*r*_1_ = 8.7 mM^−1^ s^−1^ at pH 4.0, *r*_1_ = 20.1 mM^−1^ s^−1^ at pH 7.0	pH	Fully reversible *T*_1_ contrast switch	[40]
MnOx integrated hollow MSNs	~240 nm	*r*_1_ = 8.8 mM^−1^ s^−1^ at pH 5.0, *r*_1_ = 0.8 mM^−1^ s^−1^ at pH 7.4	pH	Multimodal imaging agent (MRI and US)	[41]
Fe_3_O_4_@C@MnO_2_	130 nm	*r*_2_/*r*_1_ = 68.7 at pH 5.0, *r*_2_/*r*_1_ = 201.1 at pH 7.4	pH	Dual-modal *T*_1_/*T*_2_ contrast agent	[42]
Dopamine-responsive IONPs (DaReNa, SPIO = superparamagnetic iron oxide)	DaReNa = 138 ± 4 nm, 9D7*-SPIO = 52 ± 2 nm, Tyr-PEG-SPIO = 31 ± 1 nm	*r*_2_ = 208 ± 2 mM^−1^ s^−1^ (DaReNa), *r*_2_ = 130–140 mM^−1^ s^−1^ individual IONP species	Dopamine	Neurochemistry unaffected	[11]
Calcium-responsive nanoparticles (MaCaReNas)	35 ± 1 nm in absence of Ca^2+^, 262 ± 14 nm in presence of Ca^2+^	*r*_2_ = 151 ± 15 mM^−1^ s^−1^ with 0 mM Ca^2+^, *r*_2_ = 261 ± 21 mM^−1^ s^−1^ with 1.2 mM Ca^2+^	Calcium	Allows for calcium activity mapping in the brain	[46]
MMP-responsive iron oxide nanoparticles	Azide IONPs = 120 ± 8 nm, Alkyne IONPs = 148 ± 10 nm, presence of MMP enzymes = 780 nm	*T*_2_ signal enhancement of ~160%	Matrix metalloproteinase enzymes	Tumour-targeting contrast agent	[52]
Manganese oxide nanoparticles	8 ± 0.7 nm	*r*_2_/*r*_1_ = 14.1 in absence of superoxide radicals, *r*_2_/*r*_1_ = 31.7 in the presence of superoxide radicals	Superoxide radicals	Can mimic the enzyme superoxide dismutase, thus catalyse the dismutation of superoxide radicals	[53]
